# Renal function and biomarkers of acute kidney injury in pediatric patients

**DOI:** 10.1186/1824-7288-41-S2-A46

**Published:** 2015-09-30

**Authors:** Serafín Málaga

**Affiliations:** 1 Department of Pediatrics, University of Oviedo, Spain

## 

Acute kidney injury (AKI), previously called acute renal failure, is characterized by an abrupt increase in the concentration of serum creatinine (SCr) and nitrogenous waste products and by the inability of the kidney to appropriately regulate fluid and electrolyte homeostasis. The incidence of AKI in children has increased over the last decades, and the etiology of AKI has evolved from primary renal disease to multifactorial causes due to major advances in the medical management of critical illnesses, such as solid organ and stem cell transplantation, corrective surgery for congenital heart disease, sepsis, and septic shock. AKI is not just a marker of illness severity in children, but has a direct association with poor outcomes. Even when the definitions and characterization of AKI in children have advanced significantly over the past two decades, the diagnosis of AKI is still made with surrogate markers of glomerular filtration rate, such as SCr and urine output [1,2]. There are improved consensus multidimensional AKI definitions, namely the pRIFLE, AKIN and The KDIGO AKI definition and staging criteria being used worldwide, but with significant limitations. SCr continues to be quite limited as a marker of kidney dysfunction: it is often inaccurate in patients with low muscle mass, fluid overload or prior chronic kidney disease. Furthermore, SCr shows a demonstrable rise in concentration many hours to days after insult to the kidney, making it an insensitive, late and unmodifiable functional AKI marker. Thus, creatinine-based AKI diagnosis is often delayed, rendering treatments to mitigate or prevent AKI ineffective. There is a strong ongoing research effort to identify novel serum and urinary AKI biomarkers that reflect early kidney injury development and severity before loss of kidney function, with the hope that earlier “sub-clinical” AKI diagnosis can lead to earlier initiation of AKI treatment, or to changes in clinical management to mitigate the adverse effects of AKI until renal function recovery occurs [3,4]. Some of these promising serum and urine biomarkers are CyC and NGAL, IL-18, KIM-1, and LFABP respectively [5]. All of them have shown considerable promise diagnosing AKI earlier than SCr. Given that AKI is a complex and heterogeneous disease, it will probably be best diagnosed by a panel of biomarkers, rather than a single one. In the future, once these biomarker panels have been used widely in clinical practice and linked to clinical outcomes, it is likely that pediatric AKI will become defined by biomarker elevations and not by SCr [6].
